# Elevated CD8 T-cell counts and virological failure in HIV-infected patients after combination antiretroviral therapy

**DOI:** 10.1097/MD.0000000000004570

**Published:** 2016-08-12

**Authors:** Nam Su Ku, Awachana Jiamsakul, Oon Tek Ng, Evy Yunihastuti, Do Duy Cuong, Man Po Lee, Benedict Lim Heng Sim, Praphan Phanuphak, Wing-Wai Wong, Adeeba Kamarulzaman, Fujie Zhang, Sanjay Pujari, Romanee Chaiwarith, Shinichi Oka, Mahiran Mustafa, Nagalingeswaran Kumarasamy, Kinh Van Nguyen, Rossana Ditangco, Sasisopin Kiertiburanakul, Tuti Parwati Merati, Nicolas Durier, Jun Yong Choi

**Affiliations:** a Department of Internal Medicine and AIDS Research Institute, Yonsei University College of Medicine, Seoul, Korea; b The Kirby Institute, UNSW Australia, Sydney, Australia; c Institute of Infectious Disease and Epidemiology, Communicable Disease Centre, Tan Tock Seng Hospital, Singapore, Singapore; d Working Group on AIDS Faculty of Medicine, University of Indonesia/Cipto Mangunkusumo Hospital, Jakarta, Indonesia; e Infectious Diseases Department, Bach Mai Hospital, Hanoi, Vietnam; f Queen Elizabeth Hospital and Integrated Treatment Centre, Hong Kong, China; g Hospital Sungai Buloh, Sungai Buloh, Malaysia; h HIV-NAT/Thai Red Cross AIDS Research Centre, Bangkok, Thailand; i Taipei Veterans General Hospital, Taipei, Taiwan; j University Malaya Medical Centre, Kuala Lumpur, Malaysia; k Beijing Ditan Hospital, Capital Medical University, Beijing, China; l Institute of Infectious Diseases, Pune, India; m Research Institute for Health Sciences, Chiang Mai University, Chiang Mai, Thailand; n National Center for Global Health and Medicine, Tokyo, Japan; o Hospital Raja Perempuan Zainab II, Kota Bharu, Malaysia; p Chennai Antiviral Research and Treatment Clinical Research Site (CART CRS), YRGCARE Medical Centre, VHS, Chennai, India; q National Hospital for Tropical Diseases, Hanoi, Vietnam; r Research Institute for Tropical Medicine, Manila, Philippines; s Faculty of Medicine Ramathibodi Hospital, Mahidol University, Bangkok, Thailand; t Faculty of Medicine, Udayana University and Sanglah Hospital, Bali, Indonesia; u TREAT Asia, amfAR - The Foundation for AIDS Research, Bangkok, Thailand.

**Keywords:** cART, CD8, HIV, virological failure

## Abstract

Elevated CD8 counts with combination antiretroviral therapy (cART) initiation may be an early warning indicator for future treatment failure. Thus, we investigated whether elevated CD8 counts were associated with virological failure (VF) in the first 4 years of cART in Asian HIV-infected patients in a multicenter regional cohort.

We included patients from the TREAT Asia HIV Observational Database (TAHOD). Patients were included in the analysis if they started cART between 1996 and 2013 with at least one CD8 measurement within 6 months prior to cART initiation and at least one CD8 and viral load (VL) measurement beyond 6 months after starting cART. We defined VF as VL ≥400 copies/mL after 6 months on cART. Elevated CD8 was defined as CD8 ≥1200 cells/μL. Time to VF was modeled using Cox regression analysis, stratified by site.

In total, 2475 patients from 19 sites were included in this analysis, of whom 665 (27%) experienced VF in the first 4 years of cART. The overall rate of VF was 12.95 per 100 person-years. In the multivariate model, the most recent elevated CD8 was significantly associated with a greater hazard of VF (HR = 1.35, 95% CI 1.14–1.61; *P* = 0.001). However, the sensitivity analysis showed that time-lagged CD8 measured at least 6 months prior to our virological endpoint was not statistically significant (*P* = 0.420).

This study indicates that the relationship between the most recent CD8 count and VF was possibly due to the CD8 cells reacting to the increase in VL rather than causing the VL increase itself. However, CD8 levels may be a useful indicator for VF in HIV-infected patients after starting cART.

## 1 Introduction

Survival in HIV-infected individuals has improved since the introduction of combination antiretroviral therapy (cART).[ ^1^ ^2^] However, there continue to be individuals for whom cART fails to suppress HIV to undetectable viral load (VL) levels. ^[3]^ According to previous studies, cumulative rates of virological failure (VF) after 2 years from initial suppression ranged from 20% to 40%. [^4^ ^5^ ^6^ ^7^] Numerous predictors, such as younger age, African–American ethnicity, poor adherence to medication, missed visits, lower baseline CD4 counts, and higher baseline HIV RNA levels, have been associated with VF. [^6^ ^7^ ^8^ ^9^ ^10^ ^11^ ^12^ ^13^]

Recently, some studies have reported that elevated total CD8 counts may be a potential predictor of VF.[ ^14^ ^15^] Interestingly, a study reported that initial or serial elevated CD8 counts while on cART or an increase in CD8 counts from cART initiation may be early warning indicators of future treatment failure. ^[15]^ Also, because monitoring of CD8 counts with CD4 T cells is done in most countries, including in resource-limited settings, whether CD8 counts could be used an alternative marker of VF is important to know in terms of potential financial savings. However, these issues remain unresolved.

Thus, we investigated whether elevated CD8 counts were associated with increased risk of VF in the first 4 years of cART in Asian HIV-infected patients.

## 2 Methods

We analyzed data from the Therapeutics, Research, Education and AIDS Training in Asia (TREAT Asia) HIV Observational Database (TAHOD). ^[16]^ TAHOD is a multicenter, prospective, observational cohort study that was initiated in 2003 to assess HIV treatment outcomes in the Asia-Pacific region. ^[16]^ Patients were included in the analysis if they started cART between 1996 and 2013 with at least one CD8 measurement within 6 months prior to cART initiation and at least one CD8 and VL measurements beyond 6 months after starting cART. The analysis dataset included follow-up data collected until September 2013. We defined VF as VL ≥400 copies/mL after 6 months on cART. Elevated CD8 was defined as CD8 ≥1200 cells/μL.[ ^15^ ^17^]

Time to VF was modeled using Cox regression analysis, stratified by site. The risk analysis period began at 6 months from the start of cART and was censored at the last available VL test. CD8 counts while on cART were analyzed as a time-updated covariate lagged to the next visit. Missing CD8 observations were filled in using last observation carried forward methods (LOCF) for up to 18 months. The covariate was then coded as “unknown” after 18 months until the next measurement. Other clinical characteristics adjusted in the model were age, sex, mode of HIV exposure, pre-cART VL, CD4 and CD8, prior mono or dual therapy, initial cART regimen, hepatitis B and C coinfection, CDC disease stage, and prior TB diagnosis. Due to potential collinearity between CD4 and CD8, we did not include pre-cART CD4 and pre-cART CD8 variables in the same model. The regression models were fitted using a backward stepwise selection process. Variables significant in the univariate model at *P* < 0.10 were chosen for inclusion in the multivariate model. Variables with *P* < 0.05 in the final multivariate model were considered statistically significant. Sensitivity analyses were performed by lagging CD8 counts for 6 months as well as measuring the effects of the changes in CD8 from pre-cART values on VF.

Ethics approval was obtained from institutional review boards at each of the participating clinical sites, the data management and analysis center, and the coordinating center. All data management and statistical analyses were performed using SAS software version 9.3 (SAS Institute Inc., Cary, NC) and STATA software version 12.1 (STATA Corp., College Station, TX).

## 3 Results

In total, 2475 patients from 19 sites in China, including Hong Kong SAR, India, Indonesia, Japan, Malaysia, Philippines, Singapore, South Korea, Taiwan, Thailand, and Vietnam were included in this analysis. Of the 2475 patients, 665 (27%) experienced VF in the first 4 years of cART (Table 1). Most patients were males (78.8%) with heterosexual HIV exposure (54.9%). More than half (64.2%) initiated cART with nucleoside reverse transcriptase inhibitors (NRTIs) and non-NRTI (NNRTI) combinations. The median CD8 count prior to cART initiation was 752 cells/μL (interquartile range [IQR] 480–1089). At the time of VF, the mean VL was 82,558 copies/mL and the median was 3500 copies/mL (IQR 786–42,100 copies/mL). Additionally, there were 196/665 patients (29%) with VL 400–1000 copies/mL, 209 (31%) with VL 1001–10,000 copies/mL, 160 (24%) with VL 10,001–100,000 copies/mL, and 100 (15%) with VL > 100,000 copies/mL. However, there was no increasing trend in the proportion of patients with CD8 elevation across these VL groups (*P* = 0.357).

**Table 1:**
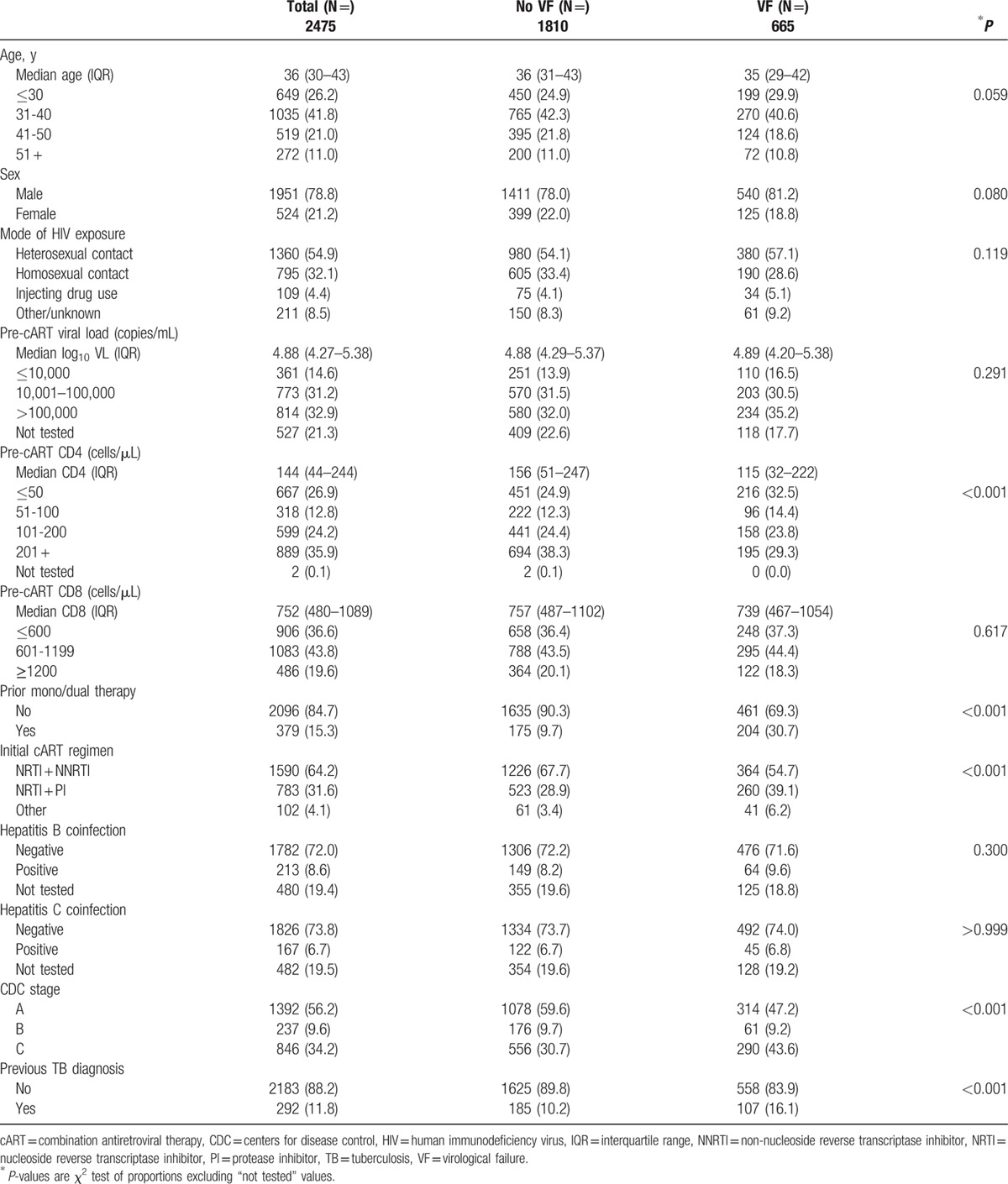
Patient characteristics.

Table 2 shows the Cox regression analysis for time to first VF in the first 4 years on cART. The overall rate of failure was 12.95 per 100 person-years. In the univariate analysis, variables that were significant at *P* < 0.10 were time-updated CD8 counts (*P* < 0.001), age (*P* = 0.005), mode of HIV exposure (*P* < 0.001), pre-cART CD4 count (*P* < 0.001), prior mono/dual therapy (*P* < 0.001), initial cART regimen (*P* < 0.001), CDC disease stage at cART initiation (*P* < 0.001), and prior tuberculosis diagnosis (*P* < 0.001). In the final multivariate model, factors significantly associated with greater hazards of VF were elevated CD8 of ≥ 1200cells/μL after cART initiation versus CD8 < 1200 cells/μL (hazard ratio [HR] = 1.35, 95% confidence interval [CI] = 1.14–1.61; *P* = 0.001), injecting drug use compared with heterosexual HIV exposure (HR = 1.74, 95% CI = 1.19–2.54; *P* = 0.005), having prior mono/dual therapy compared to initiating with cART (HR = 2.43, 95% CI = 2.01–2.95; *P* < 0.001), and being on a protease inhibitor (PI)-based regimen (HR = 1.37, 95% CI = 1.12–1.67; *P* = 0.002) and other treatment combinations (HR = 1.60, 95% CI = 1.13–2.27; *P* = 0.008) compared with NRTI + NNRTI combinations. Variables that showed a protective effect for VF were older age groups of 31 to 40 years (HR = 0.74, 95% CI = 0.61–0.90; *P* = 0.002), 41 to 50 years (HR = 0.66, 95% CI = 0.53–0.83; *P* < 0.001), 51 years and above (HR = 0.71, 95% CI = 0.54–0.94; *P* = 0.017), compared with age ≤ 30 years, and pre-cART CD4 counts > 200 cells/μL (HR = 0.68, 95% CI = 0.55–0.83; *P* < 0.001) versus CD4 ≤ 50 cells/μL.

**Table 2:**
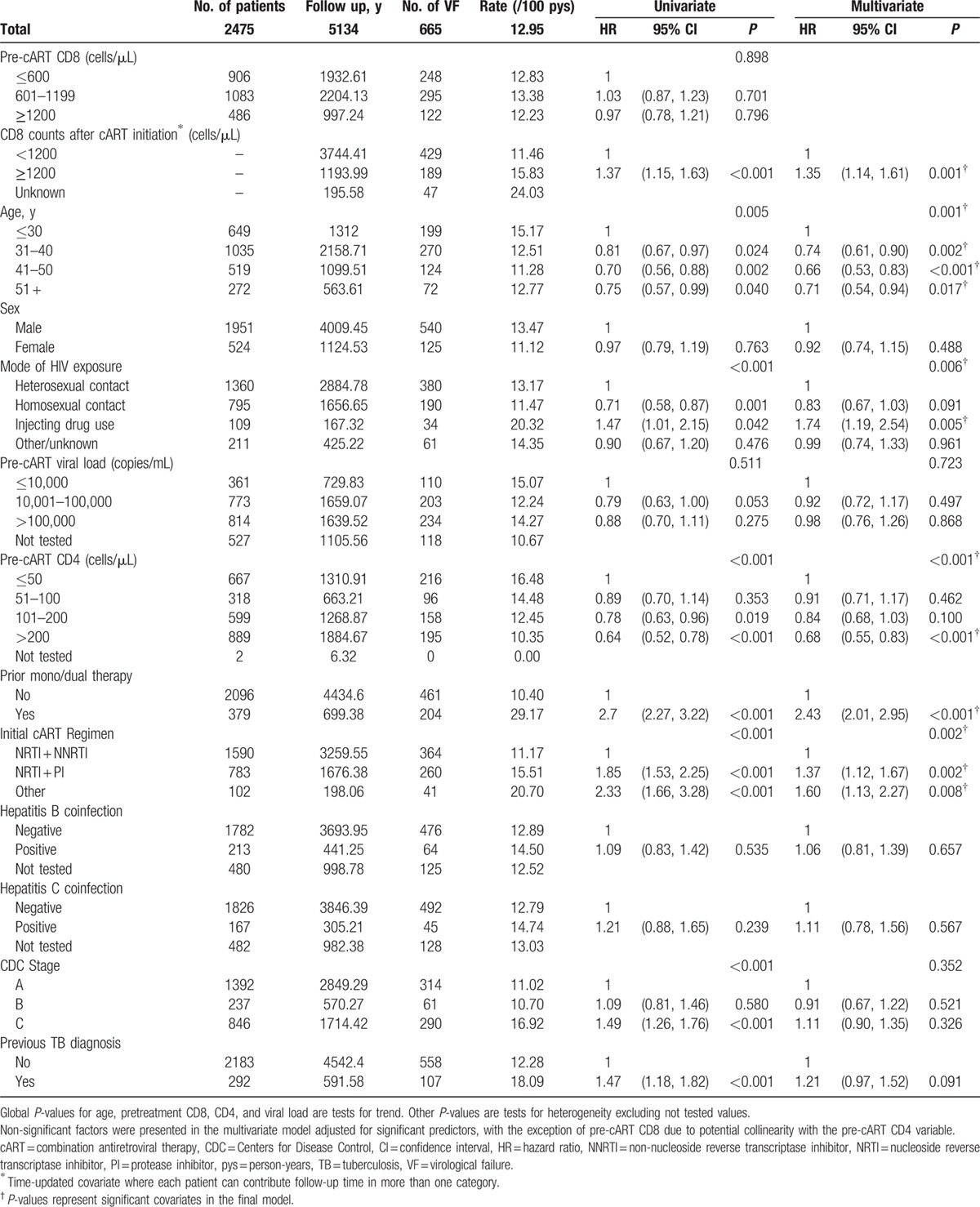
Time to virological failure.

To determine whether elevated CD8 caused subsequent VF or whether the association seen in Table 2 was the result of CD8 levels increasing due to VF, we performed a sensitivity analysis by lagging CD8 counts for 6 months. The lagging meant that VF could not be associated with CD8 measurements taken less than 6 months prior to the failure date, thus minimizing the possibility of capturing the increase in CD8 as a response to VF. Table 3(i) shows the univariate and the adjusted HRs for the lagged CD8 variable. There was no evidence to suggest that having an elevated CD8 count at least 6 months prior was associated with VF in either the univariate (HR = 1.07, 95% CI = 0.90–1.28; *P* = 0.420) or the adjusted (HR = 1.08, 95% CI = 0.91–1.29; *P* = 0.389) model. This indicates that the relationship between CD8 and VF observed in Table 2 was possibly due to the CD8 cells responding to the increase in VL rather than causing the VL increase itself. When absolute CD8 count was replaced by changes in CD8 from pre-cART values (Table 3[ii]), the increase in CD8 was borderline significant in the univariate model (HR = 1.18, 95% CI = 1.00–1.40; *P* = 0.056), but was not associated with VF in the adjusted model (HR = 1.01, 95% CI = 0.84–1.22; *P* = 0.909).

**Table 3:**
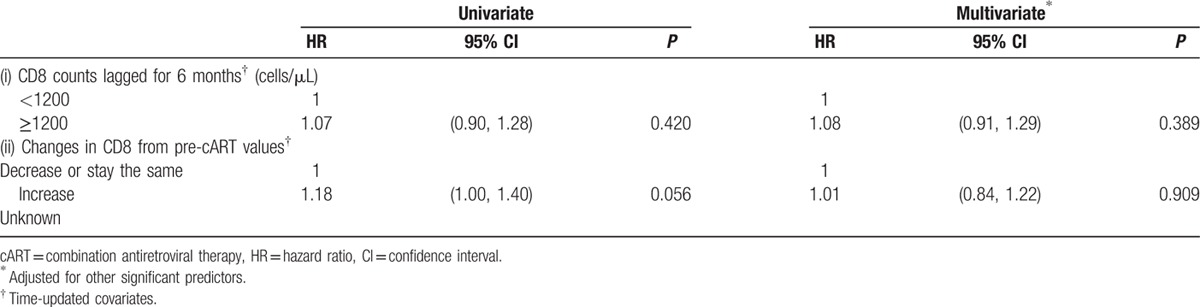
Sensitivity analyses: (i) CD8 counts lagged for 6 months, and (ii) CD8 counts replaced by changes in CD8 from pre-cART values.

## 4 Discussion

In this study, we found that the most recent elevated CD8 count after cART was significantly associated with VF, but there was no association between elevated CD8 counts measured at least 6 months prior and the subsequent virological outcome. This suggests that the CD8 levels may be considered not as a predicting factor of VF but a potential indicator for VF in HIV-infected patients after starting cART.

The relationship between CD8 response and HIV outcomes is not fully understood. CD8 response can be measured through HIV-specific CD8, CD8 activation, and total CD8 counts. ^[15]^ Among them, HIV-specific CD8 have an important role in the control of viremia, ^[18]^ increasing in response to ongoing HIV replication ^[19]^ and have been associated with poor outcomes.[ ^20^ ^21^] Activation of CD8 subsets may accelerate immune dysfunction. The expression of CD38 on CD8 has been linked to HIV disease progression. [^22^ ^23^ ^24^] One cross-sectional study showed that elevated levels of CD8 CD38 were found in HIV-infected patients with VF after cART. ^[14]^ However, it lacked sufficient sensitivity and specificity to replace viral load testing in assessing the efficacy of ART.

Additionally, some studies have demonstrated elevation of total CD8 counts in untreated HIV infection [^25^ ^26^ ^27^] and showed significant associations between elevated baseline total CD8 counts and progression to AIDS.[ ^28^ ^29^] Moreover, a retrospective cohort study recently reported that an increase in CD8 counts from cART initiation was significantly associated with treatment failure. ^[15]^ This suggests that elevated CD8 counts after cART may be useful as a predictor of VF. Krantz et al ^[15]^ explained these associations by the adverse effects of a hyperdynamic immune response or low-level viremia associated with increased risk of VF.

However, in our study, the relationship between CD8 counts and VF may be due to the CD8 cells responding to, rather than causing, the VL increase. Possible explanations for these findings include the overstimulation of CD8 immune responses against increased circulating HIV or subsequent CD8 compensation, known as “blind T-cell homeostasis,” with decreased CD4 in VF. ^[30]^ Leonard et al ^[30]^ hypothesized that in T cell loss, both CD4 and CD8 will be produced until the absolute T-cell count is normal. This suggests that the selective loss of CD4 will induce the production of both CD4 and CD8 with the result that T-cell counts will return to normal, but there would be a persistent CD8 elevation and CD4 decrease. ^[30]^

On the other hand, these findings suggest that using CD8 levels may be a potential indicator for VF in HIV-infected patients after starting cART. If CD8 levels could be used as a marker of VF, they could supplement more expensive and technologically complex VL testing. In addition, as current flow cytometer access is greater in resource-limited settings than PCR, this could facilitate more active monitoring in patient care settings. Further studies would be needed to determine the clinical and economic outcome of using CD8 levels as an indicator for VF after cART. Also, because another situation to suspect VF is CD4 count drop or failure to increase, comparing CD4 with CD8 values in terms of association with VF could be needed.

Our study limitations are primarily related to the potential for bias in our patient sampling and data. Participating cohort study sites are primarily tertiary-care referral centers, which could impact the acuity of patients as well as the level of laboratory monitoring conducted. In addition, not all TAHOD sites performed routine CD8 or VL testing, and levels of data completeness varied by both site and country. Our findings should be interpreted in these contexts, and may not necessarily apply to the broader patient populations in the Asia-Pacific. Secondly, we defined elevated CD8 as ≥ 1200 cells/μL. Although this value is the upper limit of normal, according to clinical laboratory reference values, it is based on values from Western populations.

In conclusion, our study indicates that the observed relationship between CD8 counts and VF in our cohort may have been due to CD8 cell responses to increases in VL rather than causing the increases themselves. The potential utility of CD8 levels in monitoring for VF could make it an adjunct to VL testing in the future.

## Acknowledgments

TAHOD-TASER study members:

• A Kamarulzaman, SF Syed Omar, S Ponnampalavanar, I Azwa, N Huda and LY Ong, University Malaya Medical Centre, Kuala Lumpur, Malaysia;

• BLH Sim, YM Gani and R David, Hospital Sungai Buloh, Sungai Buloh, Malaysia;

• PS Ly and V Khol, National Center for HIV/AIDS, Dermatology and STDs, Phnom Penh, Cambodia;

• E Yunihastuti, D Imran and A Widhani, Working Group on AIDS Faculty of Medicine, University of Indonesia/ Cipto Mangunkusumo Hospital, Jakarta, Indonesia;

• FJ Zhang, HX Zhao and N Han, Beijing Ditan Hospital, Capital Medical University, Beijing, China;

• JY Choi, Na S and JM Kim, Division of Infectious Diseases, Department of Internal Medicine, Yonsei University College of Medicine, Seoul, South Korea;

• M Mustafa and N Nordin, Hospital Raja Perempuan Zainab II, Kota Bharu, Malaysia;

• N Kumarasamy, S Saghayam and C Ezhilarasi, Chennai Antiviral Research and Treatment Clinical Research Site (CART CRS), YRGCARE Medical Centre, VHS, Chennai, India;

• OT Ng, PL Lim, LS Lee and R Martinez-Vega, Tan Tock Seng Hospital, Singapore;

• MP Lee‡, PCK Li, W Lam and YT Chan, Queen Elizabeth Hospital and KH Wong, Integrated Treatment Centre, Hong Kong, China;

• P Kantipong and P Kambua, Chiangrai Prachanukroh Hospital, Chiang Rai, Thailand;

• P Phanuphak, K Ruxrungtham, A Avihingsanon, C Phadungphon and S Sirivichayakul, HIV-NAT/Thai Red Cross AIDS Research Centre, Bangkok, Thailand;

• R Ditangco†, E Uy and R Bantique, Research Institute for Tropical Medicine, Manila, Philippines;

• S Oka, J Tanuma and T Nishijima, National Center for Global Health and Medicine, Tokyo, Japan;

• S Pujari, K Joshi, S Gaikwad and A Chitalikar, Institute of Infectious Diseases, Pune, India;

• S Kiertiburanakul, S Sungkanuparph, L Chumla and N Sanmeema, Faculty of Medicine Ramathibodi Hospital, Mahidol University, Bangkok, Thailand;

• TP Merati†, DN Wirawan and F Yuliana, Faculty of Medicine, Udayana University and Sanglah Hospital, Bali, Indonesia;

• R Chaiwarith, T Sirisanthana, W Kotarathititum and J Praparattanapan, Research Institute for Health Sciences, Chiang Mai University, Chiang Mai, Thailand;

• TT Pham, DD Cuong and HL Ha, Bach Mai Hospital, Hanoi, Vietnam;

• VK Nguyen, VH Bui, THD Nguyen and TD Nguyen, National Hospital for Tropical Diseases, Hanoi, Vietnam;

• W Ratanasuwan and R Sriondee, Faculty of Medicine Siriraj Hospital, Mahidol University, Bangkok, Thailand;

• WW Wong, WW Ku and PC Wu, Taipei Veterans General Hospital, Taipei, Taiwan;

• AH Sohn, N Durier, B Petersen and T Singtoroj, TREAT Asia, amfAR - The Foundation for AIDS Research, Bangkok, Thailand;

• DA Cooper, MG Law, A Jiamsakul and DC Boettiger, The Kirby Institute, UNSW Australia, Sydney, Australia.

† Current Steering Committee Chairs; ‡ co-Chairs
